# Acceptable Ungrammatical Sentences, Unacceptable Grammatical Sentences, and the Role of the Cognitive Parser

**DOI:** 10.3389/fpsyg.2020.00364

**Published:** 2020-03-10

**Authors:** Evelina Leivada, Marit Westergaard

**Affiliations:** ^1^Universitat Rovira i Virgili, Tarragona, Spain; ^2^Arctic University of Norway, Tromsø, Norway; ^3^Norwegian University of Science and Technology, Trondheim, Norway

**Keywords:** grammaticality, grammatical illusions, syntactic islands, parser, processing

## Abstract

A search for the terms “acceptability judgment tasks” and “language” and “grammaticality judgment tasks” and “language” produces results which report findings that are based on the exact same elicitation technique. Although certain scholars have argued that acceptability and grammaticality are two separable notions that refer to different concepts, there are contexts in which the two terms are used interchangeably. The present work reaffirms that these two notions and their scales do not coincide: there are sentences that are acceptable, even though they are ungrammatical, and sentences that are unacceptable, despite being grammatical. First, we adduce a number of examples for both cases, including grammatical illusions, violations of Identity Avoidance, and sentences that involve a level of processing complexity that overloads the cognitive parser and tricks it into (un)acceptability. We then discuss whether the acceptability of grammatically ill-formed sentences entails that we assign a meaning to them. Last, it is shown that there are *n* ways of unacceptability, and two ways of ungrammaticality, in the absolute and the relative sense. Since the use of the terms “acceptable” and “grammatical” is often found in experiments that constitute the core of the evidential base of linguistics, disentangling their various uses is likely to aid the field reach a better level of terminological clarity.

## Introduction

Introspective linguistic judgments about the well-formedness of linguistic stimuli have long been regarded as one of the most important sources of evidence in linguistics, essentially forming its empirical base (Wexler et al., 1975; Carr, 1990; Schütze, 1996/2016; Baggio et al., 2012). Both the techniques used to elicit such judgments (e.g., controlled experiments, self-introspection, or targeted questioning about whether a specific sentence sounds fine in a specific language) as well as the type of sample that is necessary for the results to have ecological validity (e.g., a pool of participants that is randomly selected from the targeted linguistic community, a non-random sample, or self-introspection) are a matter of debate (see Phillips, 2009; Gibson and Fedorenko, 2010; Sprouse and Almeida, 2013; Branigan and Pickering, 2016). On the other hand, no controversy exists over the fact that judgments about what forms part of a person’s linguistic repertoire constitute a rich source of information in theoretical and experimental linguistics.

Since these judgments have such a key role in the study of language, one would expect that the question of *what* they tap into would be one of the first questions in linguistics to provide an indisputable answer to. But that does not seem to be the case. If one searches PubMed or any other database for the terms “acceptability judgment tasks” and “language” on the one hand, and “grammaticality judgment tasks” and “language” on the other, one will quickly discover that the relevant experiments that will show up are the same. They all report findings that are based on the exact same elicitation technique. Perhaps the greatest illustration of how the terms “acceptability” and “grammaticality” are used, often without a clear distinction in place, comes from Schütze’s (1996/2016) seminal book on linguistic judgments. While the title of the book talks about “*grammaticality judgments* and linguistic methodology,” the very first quote given in the 2016 edition of the book is by Bever (1970), who claims that it is simultaneously the greatest virtue and failing of linguistic theory that *acceptability judgments* are used as the basic data (Schütze, 1996/2016: v). In the preface of the first edition, it is argued that “[t]hroughout much of the history of linguistics, judgments of the *grammaticality/acceptability* of sentences (and other linguistic intuitions) have been the major source of evidence in constructing grammars” (p. xi, emphasis added).

Just as linguists and other cognitive scientists have at times used the terms “ungrammatical” and “unacceptable” roughly synonymously, plurality and overlapping may characterize the use of symbols like ?, *, or ??, that are employed to signal some deviant property of the linguistic stimulus (Bard et al., 1996). To define the relevant terms, the *grammaticality* of a sentence refers to whether the sentence conforms to the syntactic rules of a given language (Fromkin and Rodman, 1998: 106), or put another way, “it is a characteristic of the stimulus itself” (Bard et al., 1996: 33). With respect to *acceptability*, the focus moves from the stimulus to a speaker’s perception; in Bard et al.’s (1996) words, it “is a characteristic of the stimulus as perceived by a speaker” (p. 33). Linguistics, however, is not a science that works exclusively with visible primitives; we cannot zoom in on a linguistic stimulus until we find and tease apart an independent, self-contained grammatical core. This means that grammaticality, as one of the possible elements that determine acceptability, “is not directly accessible to observation or measurement” (Lau et al., 2016: 3). The question thus becomes: How do we know anything about grammaticality aside of the information provided by acceptability? Put differently, if grammaticality is defined as “conforming to the rules of the grammar of language X” and if the grammar of language X has the shape that its speakers’ judgments and actual performance give it, what way do we have to capture grammaticality other than the one that goes through speakers’ perception of well-formedness (i.e., acceptability)?^1^

Answering this question is the main goal of the present work. The starting point of the discussion is Chomsky’s (1965) distinction between the terms “acceptability” and “grammaticalness,” according to which these two notions and their scales might not coincide, hence his reference to “unacceptable grammatical sentences”: sentences that do not form part of grammar for reasons that have nothing to do with grammar. The second aim of the present work is to chart the variation space that is created when one disentangles the two notions: unacceptable grammatical sentences, acceptable ungrammatical sentences, their respective parsability, and the process of assigning them meaning. Last, the scales of grammaticality and acceptability will be discussed and it will be shown that they do not coincide: there are *n* ways of unacceptability, but only two ways of ungrammaticality, in the absolute and the relative sense.

## Acceptable Ungrammatical Sentences and Unacceptable Grammatical Sentences

Humans are surprisingly good at providing accurate and consistent judgments about what forms part of their linguistic repertoire.^2^ Although informants’ opinions about their linguistic behavior are not always concordant with the way they actually speak (Labov, 1996; Cornips and Poletto, 2005), acceptability judgment tasks are reliable as a tool, and the majority of linguistic stimuli can receive unambiguous, consistent judgments. For example, little debate would occur among native speakers of English about the acceptability of (1) or the unacceptability of (2). The former is a grammatically well-formed sentence of English, while the latter is a word-salad that would probably be read and parsed in a rhythm that pertains more to lists of objects than to connected speech.

(1) John said to Mary that he likes doing linguistics.
(2) *To he likes that linguistics John Mary doing said.

Yet, even though such judgments are largely coherent with the actual shape of speakers’ internalized grammar, there are some stimuli that have the ability to trick the cognitive parser into unlawfully accepting or rejecting them. Chomsky’s (1965) discussion of “unacceptable grammatical sentences” mentions several performance-associated factors that explain why a linguistic stimulus that does not violate any rule of grammar would be rejected by speakers as unacceptable. Factors such as memory limitations, processing constraints, as well as discourse, intonational and stylistic factors may all induce such an effect. For example, overloading memory and processing resources through nested hierarchies (3) may lead the cognitive parser to not fully register or retain all the relevant information (Gibson and Thomas, 1999), something that is necessary in order to provide an acceptability judgment that faithfully represents whether the stimuli fall inside or outside the domain of predictions of the underlying, internalized grammar. In other words, precisely because of the high complexity of some stimuli, and due to the fact that the cognitive parser works on the basis of processing heuristics (Kahneman, 2011), some deviations may go unnoticed. One such example is (4), which looks very similar to (3) but–unlike (3)–violates a rule of grammar.

(3) The patient the nurse the clinic had hired admitted met Jack. Frazier (1985).
(4) *The doctor the nurse the hospital had hired met John. Frazier (1985).

In linguistic terms, the fact that (4) is missing a verb and has an argument (i.e., “the doctor”) that is not assigned any thematic role entails a violation of Chomsky’s (1981)θ-criterion, according to which each argument bears one and only one θ-role, and each θ-role is assigned to one and only one argument. Despite the seriousness of this deviation, the “missing verb effect” showed in (4) has been linked to high acceptability rates, even though the sentence is most definitely ill-formed from a syntactic point of view (Gibson and Thomas, 1999). Moreover, this effect is neither restricted to one language nor is it a laboratory phenomenon that arises only in acceptability judgment tasks (Häussler and Bader, 2015). Sentence (4) shows that ease of parsability may influence judgments, and in this specific case, low parsability leads to not spotting a violation of a core syntactic principle. At the same time, high parsability does not guarantee acceptability or grammaticality. For example, speakers of English recognize that (5) expresses a thought that their cognitive parser can easily process, but their language does not produce it in this way.

(5) *What did Peter eat ravioli and?

It seems that a dissociation is in place, because being grammatical (i.e., not violating a rule of grammar) does not guarantee acceptability either. Example (6) is in fact an unacceptable grammatical sentence.^3^ Speakers would not judge it as acceptable as (1), but it *is* a grammatically well-formed sentence of English, in the sense that no rule of grammar is violated. Its structure is analogous to that of (7).

(6) Dogs dogs dog dog dogs. Barton et al. (1987).
(7) Cats (that) dogs chase love fish.

The difficulty of (6) suggests that the types of structures that are actually attested in language are influenced by biases of general cognition. One such bias seems to underlie the unacceptability of (6): Identity Avoidance holds that elements of the same phonological and/or syntactic type are unlikely to occur in immediately adjacent positions (van Riemsdijk, 2008). Although this has long been treated as a linguistic ban, recent work has suggested that it has deeper cognitive roots, and more specifically, that it derives from the parser’s preference to avoid tokenizing multiple, adjacent occurrences of the same type because of a general bias to provide more attentional resources to novel information (“Novel Information Bias”; Leivada, 2017). Acceptability is thus affected by a variety of processing factors and cognitive biases, and so is grammaticality. For example, although data that flout Identity Avoidance exist [(6); see Leivada, 2017 for examples of syntactic violations], there are no grammatically licit structures that feature five identical, adjacent complementizers, and the prediction is that such structures will never be in use, because a grammar would never consistently deploy them. Even if grammars were able to generate a sentence like *“John said that that that that that Mary kissed him,” cognitive biases would intervene and break this sequence of complementizers, for this degree of repetition would not be informative, and by means of looking like noise to the parser, it would make communication infelicitous. A similar situation arises with sentence (4): it is extremely unlikely that a language will consistently deploy sentences with missing verbs that have licensed arguments. In other words, although the rules of the grammar of a language are subject to change in a way that may legitimize the use/acceptability of a previously ill-formed sentence and/or diminish the use/acceptability of a previously attested one, certain changes are not expected to occur, because they violate either a core principle of linguistic cognition or a general cognitive bias.

Talking about a dissociation of acceptability and grammaticality, unacceptable grammatical sentences are one logical possibility. One may wonder whether the other possibility is also attested, i.e., acceptable ungrammatical sentences. Example (8) in Table 1 provides the missing piece of this dissociation (see also Ross, 2018 for the interaction of grammaticality and acceptability).

**TABLE 1:** A dissociation of grammaticality and acceptability.

	**Unacceptable**	**Acceptable**
Grammatical	(6) Dogs dogs dog dog dogs. Barton et al. (1987).	(1) John said to Mary that he likes doing linguistics.
Ungrammatical	(2) *To he likes that linguistics John Mary doing said.	(8) *More people have been to Russia than I have. Montalbetti (1984).

*Sentences that were already introduced above appear with their original numbering.*

Sentence (8) instantiates a linguistic illusion called “comparative illusion” (Montalbetti, 1984). These sentences are called illusions because they trick the parser in a way that renders high acceptability ratings in experiments, even though the stimuli are ill-formed (Wellwood et al., 2018). In linguistic terms, (8) is ill-formed because the main clause subject calls for a comparison of cardinalities of sets, but in the absence of a bare plural in the embedded clause subject, no comparison set is made available (Phillips et al., 2011; O’Connor et al., 2012; Wellwood et al., 2018). Linguistic illusions are the outcome of a partial-match strategy that is operative during processing (Reder and Kusbit, 1991; Kamas et al., 1996; Park and Reder, 2004). When the parser receives a linguistic stimulus, its components, concepts, and structure are matched to stored knowledge, so that an output is produced. However, the parser matches the stimulus to stored information only up to a point. In other words, a processing threshold is set and the stimulus is checked up to this threshold, hence the notion of partial matching. Given that (8) makes use of locally coherent templates (Townsend and Bever, 2001) that provide a “good-enough fit” (Ferreira and Patson, 2007) for the parser, its ill-formedness may go unnoticed, and this results in high acceptability. Evidently, the way the parser works–via the use of processing heuristics–mediates one’s access to the internalized knowledge of grammar. Yet, the ease with which a sentence is unambiguously parsed is not a guarantee for either grammaticality or acceptability. Table 2 adds high/low parsability to the previous dissociation between grammaticality and acceptability. Once again, all logical possibilities are attested.

**TABLE 2:** A dissociation of grammaticality, acceptability, and parsability.

	**High parsability**	**Low parsability**
Grammatical/acceptable	(1) John said to Mary that he likes doing linguistics.	(3) The patient the nurse the clinic had hired admitted met Jack. Frazier (1985).
Grammatical/unacceptable	(9) Colorless green ideas sleep furiously.^1^ (Chomsky, 1957)	(10) That that that Bill left Mary amused Sam is interesting is sad. Hornstein (2013).
Ungrammatical/unacceptable	(5) *What did Peter eat ravioli and?	(2) *To he likes that linguistics John Mary doing said.
Ungrammatical/acceptable	(11) *Fewer people have been to Tromsø than I have.	(4) *The doctor the nurse the hospital had hired met John. Frazier (1985).

*Sentences that were already introduced above appear with their original numbering. ^1^The grammaticality of (9) is fairly indisputable (Hill, 1961), however, not everybody agrees on the degree of its unacceptability. Some scholars have talked about doubtful acceptability, marking the sentence with a “?” to indicate this (Armstrong, 2005), while others have described it as outright unacceptable (Bauer, 2014).*

Example (9) does not violate any rule of grammar, however, its acceptability is not comparable to that of (1) for semantico-pragmatic reasons that boil down to difficulties that arise “in assigning a coherent meaning to the whole” (Adger, 2018: 161). Unlike (2) or even (10), (9) can be easily parsed in a way that pertains to connected speech. Moreover, a coherent interpretation of it can be provided, and over the years there have been various proposals that construe meanings for it.^4^ Perhaps green ideas refer to environmental considerations. One could build a metaphorical narrative where these ideas are colorless and sleeping because at present there is not enough effort to combat climate change, however, their sleep is furious, something that may suggest that some promising initiatives for change are under way. Creating the right context can improve the acceptability of (9) precisely because of its grammatical well-formedness and high parsability.

Perhaps the most interesting sentence of Table 2 is (4): a sentence that is both ungrammatical and hard to parse, yet still acceptable. Its low parsability hides the grammatical violation, something that leads to high acceptability. Of course, one could claim that such a sentence, despite being labeled “acceptable,” would never be attested in one’s linguistic performance. However, ungrammatical sentences that are harder to parse *are* in fact attested in naturalistic speech (12a), and the relevant data also include missing verbs in cases of center-embedding (12b).

(12a) “And since I was not informed–as a matter of fact, since I did not know that there were excess funds until we, ourselves, in that checkup after the whole thing blew up, and that was, if you’ll remember, that was the incident in which the attorney general came to me and told me that he had seen a memo that indicated that there were no more funds.”^5^ President Ronald Reagan, April 28, 1987.
(12b) That we scrutinize is a simple consequence of the fact that none of the predictions that you ****Δ**** during the months that you have been in office has turned out to be true. Häussler and Bader (2015: 14).

Going back to the rest of the data in Table 2, we see that (5) and (11) suggest that certain ungrammatical sentences can be easily parsed too. Recent research has suggested that not all ungrammatical sentences receive unclear and unreliable interpretations across speakers (e.g., Etxeberria et al., 2018 on negation). Talking about ungrammatical sentences that are acceptable and parsable, Otero (1972) reached the conclusion that wide acceptability is not a guarantee for grammaticality. Even sentences that have been described as blatantly ungrammatical may actually be acceptable to some degree, and this degree varies across speakers of the same language that have different developmental trajectories (e.g., late bilinguals, heritage speakers, L1 attriters). For example, (5) is ungrammatical because extraction out of coordinated structures is prohibited. A similar island effect has been described for extraction out of relative clauses (13).

(13) *Who do you like the poem that____wrote?

Although much literature portrays such sentences as universally ungrammatical (see Phillips, 2013 and references therein), not all speakers find such violations fully unacceptable. For instance, Lowry et al. (2019) found surprising rates of acceptability for five different types of island violations–including relative clause islands that received a mean score of 3.6 in an 1–5 scale, where 1 stood for the sentence sounding perfectly natural–among late bilingual and heritage speakers of Spanish. Importantly, the two groups differed both in terms of their judgments and in terms of their involuntary physiological reactions that can be proxies for processing effort. In Lowry et al. (2019) these were measured through a pupillometry study: pupil dilation in the ungrammatical stimuli was observed only in the group of late bilinguals, while there was no effect of ungrammaticality in the heritage group. These results suggest that regardless of what a theory/grammar presents as ungrammatical, speakers may successfully parse ungrammatical stimuli in a way analogous to their grammatical counterparts. However, it is an important question whether the parsing is complete, in the sense that these speakers assign meaning to these ungrammatical stimuli.

Understanding the process of assigning meaning is important in the context of disentangling the role of the parser in acceptable ungrammatical sentences. To illustrate this, let’s consider the comparative illusions in Tables 1, 2 [examples (8) and (11), respectively], which are ungrammatical but trick the parser into acceptability (Wellwood et al., 2018; Leivada et al., 2019b). Although various experiments have shown that these sentences are assigned a high acceptability rating, one could say that this does not entail that these sentences are actually parsed, in the sense that speakers actually assign them a meaning *m*. A clear exposition of this point is given by Tim Hunter as a reply to Hornstein (2013), who suggests that such sentences may sound good to speakers, but when you ask the people that gave them a high rating what the uttered sentence means, they are unable to provide a meaning:

I don’t think there is any meaning *m* such that (“More people have been to Russia than I have,” *m*) is judged acceptable. What **is** true about these examples is that if you ask whether the string is acceptable without providing any intended interpretation–roughly, if you ask a question of the form “Is there a meaning *m* such that (*s*, *m*) is acceptable?”–then people tend to say “yes.” This despite the fact that, as everyone points out, if you ask which meaning this is, people are stumped. […] Why they should make this kind of mistake (i.e., accept the sentence), I have no idea: presumably the answer might be something like, they start searching for a meaning for the string, and they get close enough to feel confident that a meaning **can** be found without getting all the way there, so they stop and answer “yes” (since no one is asking for the particular meaning).

In contrast, we suggest that illusions like (8) and (11) are parsed in a way that does go through assigning *m* to *s*. In our work on comparative illusions (Leivada et al., 2019b) we obtained ample evidence that most speakers that judged (8) as acceptable, truly construed an interpretation for it. Among the ones more frequently given by the speakers we tested are: (i) more people than just me have been to Russia, (ii) people have been to Russia more times than I have, and (iii) many people have been to Russia more times than I have (see also Wellwood et al., 2018). Naturally, this is not what the sentence says, but nevertheless, a meaning *is* assigned to the sentence. Also we suggest that one should not ignore the possibility that those speakers that seem stumped upon being asked to provide an interpretation do not do so because they never actually established an association (*s*, *m*), but because in their attempt to articulate the latter, they spot the illusion. Crucially, this does not entail that at no point were they actually able to put their finger on a possible meaning.

The second interesting issue with Hunter’s point has to do with the juxtaposition of two very different ways of eliciting judgments through asking “Is *s* acceptable?” or “Is there a meaning *m* such that (*s*, *m*) is acceptable?” These two questions do not tap into the same thing. Previous research on the pragmatics of cognitive illusions has proposed that when processing such sentences, the hearer searches for meaning within a manipulative communication, that is, within a tricky context that features a “manipulation (that) can be best defined in terms of the constraints it imposes on mental processing” (Maillat and Oswald, 2009: 361). In this context, the hearer stops searching for meaning after finding one that sufficiently meets her expectations of relevance in accordance with the previous discourse. The illusion thus arises in the process of selecting meaning within a manipulative context that takes advantage of (i) the parser’s limitations and (ii) the parser’s way of operating through employing certain processing heuristics such as partial matching or shallow processing.

If relevance and previous context can bias an acceptability judgment through creating the necessary conditions for an illusion to arise, the bias will be even greater if a specific *m* is given to a participant point-blank in an experiment that asks “Is there a meaning *m* such that (*s*, *m*) is acceptable?” As shown in Tversky and Kahneman’s (1974) work, options in a task are evaluated relative to some *reference point*. Theoretically speaking, the reference point in standard acceptability judgment tasks is the linguistic repertoire of the tested speaker: We often instruct speakers to disregard the formal prescriptive rules of grammar and focus on evaluating the stimuli on the basis of how they use the language. If we add a given *m* to this picture, we alter the reference point. This does not mean that such a task cannot provide useful and informative findings, but that possibly the obtained findings will not be tapping directly into a speaker’s perception of her idiolect. Instead, it will be mediated by an *anchoring effect* that may cause an *adjustment* to the speaker’s judgment on the basis of *m*. To understand this effect, consider the following example by Kahneman (2011).

(14a) Was Gandhi more or less than 144 years old when he died?
(14b) How old was Gandhi when he died? Kahneman (2011: 122).

Of course nobody claimed that Gandhi was 144 years old when he died, but it has been found that when (14b) is presented after (14a), the provided high number functions as an anchor that affects people’s estimate (Kahneman, 2011). To draw the analogy with judgment tasks, let’s compare (15a) to (15b), and it will become clear why “Is *m* acceptable?” does not ask the same thing as “Is there a meaning *m* such that (*s*, *m*) is acceptable?.”

(15a) Assuming a scale from 1 to 5, how acceptable is *s* on the basis of an intended meaning *m*?
(15b) Assuming a scale from 1 to 5, how do you rate *s* on the basis of your idiolect?

In (15a), the possibility of *s* getting a meaning is explicit and a possible meaning *m* is already given to the speaker as part of the question that introduces the stimuli *s*. This can bias the rating of *s* on the basis of the “anchor-and-adjust” heuristic.

To sum up, illusions do not necessarily entail that parsing fails to produce a meaning, but that the parser can be tricked into providing both a meaning and an acceptability rating that may not correspond to the actual status of the stimulus in terms of what the speaker’s internalized grammar looks like. Importantly, a number of factors contribute to this process of tricking the parser: context, task and stimuli presentation, as well as structural complexity are only a few.

The relation between grammatical well-formedness and acceptability is a complex one. As mentioned in the Introduction, the main goal of the present work is to discuss whether acceptability is an indispensable gateway to grammaticality or whether there is a way of capturing grammaticality other than the one that goes through speakers’ perception of what is well-formed in their native linguistic repertoire (i.e., acceptability). Having presented the dissociation between acceptability, grammaticality and the way the parser works, the next section deals with how grammaticality is established and where it comes from.

## Where Does Grammaticality Come From?

Asking about the origin of the rules of grammar, Adger (2019) suggests that we learn them: They come from the way people speak. Although this is true, the issue is more complex, because different people speak in different ways even within linguistic communities that feature only one language. When one says that (5) and (8) are ungrammatical in English, this use of the term “ungrammatical” is not meant to be interpreted as a faithful representation of every English speaker’s idiolect in an individual way, precisely because even monolingual speakers in a monolingual community show variation.^6^ Rather it refers to some established consensus about what is the norm in a specific variety of English; a norm that the grammar books describe in detail. Put differently, if some speakers of English, Spanish, or German accept to some degree or even produce to some degree island violations (Lowry et al., 2019 for Spanish), missing verbs in nested hierarchies (Häussler and Bader, 2015 for English and German), or comparative illusions^7^, do we want to say that these structures are grammatical in English, Spanish, and German? While it certainly appears to be the case that some speakers’ grammars may occasionally give rise to such structures, we should take into account that, in relation to naturalistic data, production factors may endow the linguistic message with noise (i.e., false starts, infelicitous lemma retrievals, missing elements due to memory constraints, etc.), which can account for how some of these ungrammatical sentences come to be produced in spontaneous speech. In relation to the possible acceptability of these structures in experimental settings, the previous section has shown that there is a dissociation between acceptability and grammaticality, such that we should expect some degree of discrepancy between the way speakers judge sentences in an experiment (where even the way the stimuli are presented may influence judgments; see examples 14–15), the way they actually speak, and the way that prescriptive grammar says they (should) speak.

The question still holds: Where does grammaticality come from? The tentative answer we offer is that grammaticality is often a formal, standardized snapshot of the way the official language looks like at a given point in time. Grammaticality is constantly redefined through ever-changing acceptability, but it also reflects stable properties of general cognition. In this context, we do not know much about grammaticality outside acceptability (recall that observation of naturalistic data cannot reveal what is ungrammatical in a language) in the sense that there is no list of grammatical properties that are grammatical *in and of themselves*. They are all grammatical within a context that is called language X. Language X is constantly changing and what is (un)grammatical today may not be (un)grammatical tomorrow, depending on whether the new speakers of X find it acceptable or not and whether this acceptability is generalized and established as the norm or not. For example, Ancient Greek featured a syntactic phenomenon called Attic syntax which permitted a number mismatch between the plural, neuter subject and the verb (16a). This structure is not a grammatically licit option in Modern Greek (16b), but not because there is something intrinsically *un*grammatical about it; it simply does not form part of the grammar anymore. Phrased differently, there is no notion of *self-contained* grammaticality that (16a) has and (16b) lacks; they just form part of two different snapshots of a grammar’s domain of predictions at different points in time.

(16a) Ta padia pezi. [Ancient Greek]
  the child.PL play.3SG
  “The children are playing.”
(16b) *Ta koritsia gela. [Modern Greek]
  the girl.PL laugh.3SG
  Intended meaning: “The girls are laughing.”

This claim is partially concordant with Chomsky et al.’s (2019); see also Chomsky (1993) view that in natural language there exists no *independently given* notion of grammatical well-formedness. Indeed, the grammatical well-formedness of a linguistic stimulus does not boil down to an independently definable grammatical core, but is a mere historical “accident” that (i) refers to whether the stimulus forms part of the standardized snapshot or not and (ii) is subject to change such as the one shown in (16a-b). Nevertheless, this view is true only for one reading of the term “grammatical”: grammatical as *actually forming part of the grammar of a specific language*.

However, we suggest there is also another reading of the term “grammatical.” To understand this other reading, one needs to factor in that change is not without limits. Not all changes are possible and not all linguistic stimuli are candidates for forming part of grammar. For example, as mentioned in the section “Acceptable ungrammatical sentences and unacceptable grammatical sentences” there are no grammatically licit structures that feature five identical, adjacent complementizers, and the prediction is that such structures will never be grammatical. Similarly, a sentence such as (4), which violates the θ-criterion, is unlikely to ever form part of grammar.^8^ As discussed in the next section, certain changes are not expected to occur, because they violate either a core principle of language (e.g., the θ-criterion in 4) or a general cognitive bias (e.g., the Novel Information Bias in 6). In this sense, Chomsky et al. (2019) are right in arguing that there exists no independently given notion of grammatical well-formedness, but we would like to add to their claim that there do exist independently given constraints to the set of entities that this notion can encompass. This is the other reading of the term: grammatical *as having the potential to be a part of grammar*, by means of not going against any of the relevant biases and communication/processing principles that underlie language and cognition.

## *N* Types of Unacceptability and Two Types of Ungrammaticality

It is an uncontroversial claim that acceptability judgments are not categorical, but form a continuous spectrum (Sprouse, 2007 and references therein). The usual meaning of the word “continuous” is *unbroken* or *undivided*, hence it is the nature of a continuum to be undivided, or better, to permit repeated division without limit (Bell, 2017). If one subscribes to the view that acceptability should be viewed as a continuum, one also subscribes to the view that the acceptability continuum is *infinitely divisible*. Although acceptability judgment tasks that involve Likert scales feature a finite number of options more often than not, there are experiments that ask speakers to judge a linguistic stimulus by adjusting a slider on a continuum without any clearly delineated categories such as “acceptable,” “somewhat acceptable” etc.

While the scale of unacceptability involves *n* positions, the scale of ungrammaticality involves only two: Something can be ungrammatical in the *relative* or in the *absolute* sense. The relative sense pertains to the first reading of the term “grammatical” that was mentioned above: forming part of grammar. We call it “relative” because it is defined in the context of a given language. For example, (16b) is ungrammatical *in relation to* Modern Greek, but it is not ungrammatical *per se*. It is a potential candidate for forming part of grammar, it was grammatical in the past (16a), and it may be again in the future. Similarly, (17) is ungrammatical in relation to Standard English, but this is an accident, as it could potentially be grammatical (and in fact it is grammatical in many varieties of English, including e.g., Belfast English; Henry, 2005).

(17) The children is here.

Relative ungrammaticality (i) is subject to change, (ii) is defined in the context of a specific language, and (iii) refers to those sentences that could be grammatical, but for some reason are not in the language in question, yet they probably are in some other language. Absolute ungrammaticality (i) is not subject to change, (ii) is not defined in relation to one given language, and (iii) concerns violations of some core principle of language and/or cognition, that is, structures that grammar would never consistently deploy. Therefore, absolute ungrammaticality has to do with structures that *cannot* form part of grammar.

Comparing the scales of the two notions, acceptability and grammaticality, it is meaningful to talk about partial acceptability (Sprouse, 2007), but not about partial or strong-weak ungrammaticality. A rule of grammar (or more than one rule of grammar) can be either violated or not, but it cannot be violated just a bit. Ungrammaticality cannot be a matter of degree, only acceptability can. Put differently, a native speaker can judge a structure in her language as more acceptable than another structure, but a structure forming part of a grammar cannot be more grammatical than another structure that forms part of the same grammar.

Although some scholars have talked about “partial ungrammaticality,” we would argue that this refers either to partial unacceptability or to variation in a linguistic community. Consider, for instance, the discussion of partial ungrammaticality in Attinasi (1974): “A hidden assumption of homogeneity, that the language competence of every speaker consists of the same structures, falters when the question of partial ungrammaticality is raised. How can some speakers totally reject, others partially accept and still others totally accept certain sentences as grammatical if each presumably speaks ‘English,’ or any other language?” (p. 280). In our view, this question has to do with gradient *acceptability*: that is what speakers have judgments about.^9^ As we have seen, grammaticality can be dissociated from acceptability. Also, the observed variation does not entail or legitimize the notion of partial grammaticality, because, as mentioned in the section “Where does grammaticality come from?,” different people speak in different ways, but grammaticality evokes an established norm that is part of a formal snapshot. Speakers may deviate from this norm, either because language change has occurred and the norm does not reflect this yet, or because their idiolect simply differs from the norm. But this should be referred to as “interspeaker variation,” not “partial grammaticality.”

## Outlook

The present work has discussed the complex relation between grammaticality, acceptability, and parsability. A number of unacceptable grammatical sentences and acceptable ungrammatical sentences have been presented, including grammatical illusions, violations of Identity Avoidance, and sentences that involve a high level of processing complexity that overloads the cognitive parser. Focusing on acceptable ungrammatical sentences, we have argued that in many cases their acceptability entails that a meaning has been assigned to them. Also, two notions of ungrammaticality have been introduced: (Un)grammaticality in the relative sense refers to the whether the stimulus falls within the domain of predictions of a given grammar or not. (Un)grammaticality in the absolute sense refers to whether the stimulus has the potential to be a part of grammar or not. Relative (un)grammaticality is an ever-changing property of the stimulus, whereas absolute (un)grammaticality is stable. In both readings of the term, grammaticality is defined by something that is external to the stimulus (be it the grammar of a specific language or principles of general/linguistic cognition), and it is not an inextricable property of the stimulus itself. Put differently, there is no list of properties that are (relatively/absolutely) grammatical *in and of themselves*, or as Chomsky et al. (2019) phrase it, there is no independently given notion of grammatical well-formedness in natural language.

Through disentangling the various uses of the terms “acceptable” and “grammatical,” the overarching aim of this work has been to aid the field in reaching a more adequate level of terminological clarity for notions that pertain to the evidential base of linguistics. Many details of the distinction between relative and absolute (un)grammaticality are left to be worked out, and this will likely be the topic of future work. To give just one example, when we deal with island effects of the sort discussed above, do we deal with absolute ungrammaticality that is universal and derives from processing or other principles of language/cognition, or with relative ungrammaticality that is manifested in different ways across different languages, precisely because it is defined on the basis of language-specific factors? Or as Ott (2014): 290) asks is “*What does John like and oranges?” ungrammatical (in the absolute sense that *it cannot be generated by the grammar*), given that speakers can easily assign it a transparent interpretation (e.g., which *x*: John likes *x* and oranges)? The answer is currently unclear to us, and it probably needs novel experimental work to be properly discussed. Recognizing this uncertainty does not mean undermining the proposed distinction between absolute and relative ungrammaticality. It rather suggests that progress is underway, or as (Feynman, 1998: 27) puts it, “[b]ecause we have the doubt, we then propose looking in new directions for new ideas. The rate of the development of science is not the rate at which you make observations alone but, much more important, the rate at which you create new things to test.”

## Author Contributions

EL and MW conducted the research behind this work. EL drafted a first manuscript, which MW revised. Both authors contributed equally to the final editing of this work.

## Conflict of Interest

The authors declare that the research was conducted in the absence of any commercial or financial relationships that could be construed as a potential conflict of interest.

## Footnotes

1 An obvious answer could be that rules of grammar could be extrapolated through corpora of naturalistic speech. Although such corpora are useful, they cannot substitute judgments, for two reasons. First, they are informative only about what is part of a language, but cannot show the actual limits of variation. It is impossible to establish what is *not* licit in a language only by analyzing them (Henry, 2005). Second, big corpora with rich data that include a variety of genres are the only ones that can provide a faithful approximation of the actual variation space of a language, and these are available only for big, standard languages. This is one of the most important challenges that linguists working with small or non-standard languages face (Leivada et al., 2019a). For these reasons, native judgments are an indispensable tool for most linguists.

2 This is important because accuracy and stability of judgments are not present in *all* types of judgments that are related to some aspect of human perception. For example, in the famous “The Dress” photograph, not only did judgments of color perception differ across people, with some seeing the dress as blue/black and others as blue/brown or white/gold, but also test-retest reliability revealed switches in perception across testing sessions (Lafer-Sousa et al., 2015).

3 “Dog” can be both a verb and a noun in English. The sentence means the following: dogs that are followed by dogs follow themselves other dogs.

4 https://www.physicstomato.com/colorless-green-ideas-sleep-furiously/

5 http://www.reagan.utexas.edu/archives/speeches/1987/042887e.htm

6 For example, Smith and Cormack (2002) discuss sequences of tense possibilities in English. With some speakers accepting “Did you know that Emily is ill?” and with others considering it unacceptable (i.e., accepting only “Did you know that Emily was ill?”), these authors capture the observed variation by suggesting that this is “a situation in which intuitions are completely clear-cut, so the relevant parameter has been fixed, but it has been fixed apparently at random, presumably because of the paucity of distinguishing data” (p. 286). Another example is given in Levelt (1972), who showed that opinions about what is grammatical in a language are not uniform even among trained linguists who are native speakers of the language in question. When he asked 24 linguists to judge whether the sentence “The talking about the problem saved her” (Fraser, 1970, p. 91, with the example marked as ungrammatical) was marked as grammatical or ungrammatical in a specific linguistics article, he found that judgments varied, and only 1/3 of the consulted linguists gave the judgment “ungrammatical,” in agreement with the original source.

7 One example of a comparative illusion in naturalistic speech, outside of an experimental setting, is the following tweet by Dan Rather: “I think there are more candidates on stage who speak Spanish more fluently than our president speaks English.” [Available at https://twitter.com/danrather/status/1144076809182408704]

8 Although the missing-verb effect can be occasionally attested in naturalistic speech (12b), we argue that this has to do with production factors that introduce noise to the linguistic message.

9 Boeckx (2010) rightly calls the term “grammaticality judgment tasks” a misnomer, because speakers lack intuitions about whether something is grammatical. In the absolute meaning of the term “grammatical,” having judgments about grammaticality would entail having intuitions about the workings of *all* linguistic and cognitive factors that determine the limits of grammar, and no speaker (or linguist for that matter) has that.
